# The impact of medication side effects on adherence and persistence to hormone therapy in breast cancer survivors: A qualitative systematic review and thematic synthesis

**DOI:** 10.1016/j.breast.2021.05.005

**Published:** 2021-05-17

**Authors:** Nicola Peddie, Sommer Agnew, Megan Crawford, Diane Dixon, Iain MacPherson, Leanne Fleming

**Affiliations:** a University of Strathclyde, UK; b University of Aberdeen, UK; c University of Glasgow, UK

**Keywords:** Adjuvant hormone therapy, Breast cancer, Side effects, Endocrine therapy, Tamoxifen, Aromatase inhibitor, Adherence, Persistence, Qualitative

## Abstract

**Background:**

Hormone Therapy (HT) reduces the risk of breast cancer recurrence and mortality in women with breast cancer. Despite these clinical benefits, rates of HT non-adherence and non-persistence are high. Research suggests this may be due to the impact of HT side effects. However, little research has explored the individual contribution of side effects to non-adherence and non-persistence behaviours, thereby hindering the implementation of targeted intervention strategies. Our aim is to review the published literature on breast cancer survivors’ lived experiences of HT side effects and explore how these may be related to non-adherence and non-persistence behaviour.

**Methods:**

Electronic searches were conducted from inception to May 2020, utilising Cochrane CENTRAL, Medline, Embase, Web of Science and PsycINFO databases. Searches included a combination of terms related to breast cancer, adherence, hormone therapy and side effects.

**Results:**

Sixteen eligible papers were identified, and study quality was high. Data were thematically synthesised into four analytical themes, which encompassed 13 descriptive sub-themes: ‘Daily impact of side-effects’, ‘Role of Health Care Professionals’, ‘Managing HT side-effects’, and ‘Weighing up the pros and cons’.

**Conclusions:**

HT side effects significantly impact breast cancer survivor's quality of life. A lack of support from healthcare providers leads to self-management strategies, which negatively affects adherence and persistence behaviour.

## Highlights

• HT reduces risk of recurrence, yet many cancer survivors do not take HT as prescribed.
• Adherence to HT is negatively impacted by the presence and severity of side effects.
• 16 studies were synthesised to explore how side effects impacted adherence and persistence.
• HT side effects significantly impact breast cancer survivor's quality of life.
• Healthcare professionals play an important role in encouraging adherence and persistence.

## Abbreviations

**List of Abbreviations used**
AI: Aromatase Inhibitor
HCP: Health Care Professional
HT: Hormone Therapy

## 1 Introduction

Breast cancer is the most common cancer in the UK, accounting for 15% of all new cases and is the third most common cause of cancer-related deaths [1]. Hormone Therapy (HT), is a treatment that suppresses hormone production or that interferes with hormone receptor signalling in order to prevent tumour growth, is prescribed to breast cancer survivors for up to ten years following diagnosis. Around three quarters of breast cancers are hormone-receptor-positive and are treatable with HT. The most commonly prescribed HTs are Selective Estrogen Receptor Modulators (SERMS) (e.g. Tamoxifen) and Aromatase Inhibitors (AI) (e.g. Letrozole, Anastrozole and Exemestane) [2]. When taken as prescribed, HT reduces the risk of breast cancer recurrence by 40% and mortality by a third [3]. However, despite its clinical efficacy for recurrence prevention, many cancer survivors do not take their HT as prescribed. Approximately 50% of women take less than 80% of the prescribed dosage [4] and up to 50% discontinue their treatment by the fifth year of prescription [5]. This pattern of poor persistence and non-adherence is associated with increased odds of breast cancer recurrence and mortality [6]. Adherence to and persistence with hormone therapy is therefore considered a key determinant of disease-free survival.

Adherence is defined as the degree to which an individual's behaviour corresponds with agreed treatment recommendations in terms of dose, timing and frequency, whereas persistence refers to the duration of treatment from initiation to discontinuation [7]. Recent research has begun to explore the mechanisms that impact upon adherence and persistence behaviour and has identified sociodemographic, clinical and psychosocial characteristics as potential risk factors [8,9]. Sociodemographic variables such as age, socioeconomic status, ethnicity, education and out of pocket costs of medications have been shown to negatively impact HT adherence and persistence [[10], [11], [12]]. Similarly, clinical characteristics such as switching between treatments (e.g. from Tamoxifen to Letrozole) is also negatively associated with adherence and persistence [12]. Whilst the identification of sociodemographic and clinical risk factors facilitate our understanding of those individuals most likely to be non-adherent and non-persistent, they have limited value when considering strategies to promote positive behaviour change. However, identifying psychosocial characteristics that increase the risk of non-adherence and non-persistence to HT does provide a potential pathway to behaviour change interventions to promote adherence and persistence.

In a recent review of barriers and facilitators of HT adherence and persistence [9], several factors were identified as possible intervention targets due to their impact on adherence and persistence behaviour. One of these factors was categorised as ‘side effects from HT’ and included cognitive, gynaecological, musculoskeletal and sleep/fatigue-related symptoms. Similarly, numerous studies have found that patients' experience of HT side effects (joint pain, hot flushes, night sweats, fatigue), affect adherence and rates of treatment discontinuation [8,13,14], potentially because the adverse effects of treatment outweigh the perceived benefits [15]. Therefore, unlike sociodemographic and clinical factors that are not amenable to change, side effects are suggested intervention targets because their effective management has the potential to increase long-term HT adherence and reduce rates of treatment discontinuation. However, the contribution of specific side effects to HT non-adherence and non-persistence is poorly understood, making the development and prioritisation of targeted intervention strategies difficult. Many studies prefer instead to utilise quantitative questionnaire-based methodologies to report an overall side effect profile where presence/absence of side effects are reported as a single yes/no variable [[16], [17], [18]]. In addition, a lack of consistency in published terminology (e.g. adherence, compliance, persistence, continuance), definitions (e.g. medication possession ratio (MPR) of ≥80% or discontinuation before 5 years), data measurements and sources (e.g. medical records, clinician checklists, self-report questionnaires) make cross-study comparison challenging [19]. Therefore, greater consistency and qualitative understanding of the impact of individual side effects would help to identify and quantify the specific psychosocial determinants of non-adherence and non-persistence that are modifiable through intervention.

Two recent reviews of qualitative research in this area [19,20] have identified that adherence to HT is negatively impacted by the presence and severity of side effects. However, these reviews were broad in scope, considering multiple patient-related factors (e.g. quality of life, self-efficacy, patient beliefs, side effects) that influenced adherence and persistence. Therefore, whilst these studies provide valuable insight into the lived experience of taking HT, further qualitative work must explore the primary motivations for non-adherence and non-persistence if tailored approaches for managing side effects are to improve adherence and persistence behaviour in breast cancer survivors. Therefore, the aims of this review are to (i) synthesise breast cancer survivors’ lived experiences of HT side effects and (ii) conduct an in-depth exploration of how these experiences may influence adherence and persistence behaviour. By synthesising experiences of side effects and how they are perceived to influence adherence and persistence behaviour, potential facilitators and barriers to adherence and persistence will be identified. This will inform the implementation of targeted evidence-based interventions to improve adherence to HT and reduce rates of recurrence and mortality.

## 2 Method

This review has followed the ENTREQ statement [21] and has adopted Thomas and Harden's (2008) [22] approach to thematic synthesis.

### 2.1 Search strategy

A comprehensive search strategy was developed and adapted from Moon et al. (2017) [9]. A combination of search terms related to 1) breast cancer, 2) adherence and persistence 3) hormone therapy and 4) side effects were included. A full copy of the search strategy is included as a supplementary file. Electronic searches were performed from inception to May 2020 using the following databases: Cochrane CENTRAL, Medline, Embase, Web of Science, and PsycINFO. The review protocol was registered on the PROSPERO database on August 13, 2020 (CRD42020192481). As outlined in the protocol, we originally planned to conduct a mixed-methods review of the literature on the impact of HT side effects on adherence and persistence. However, the initial search generated such a significant volume of quantitative and qualitative studies that it was decided to be more valuable to publish two separate reviews.

### 2.2 Inclusion criteria

Studies were included if they utilised a qualitative methodology to investigate the impact of HT side effects on adherence and/or persistence in adult, female breast cancer survivors. No exclusion criteria were applied to year of publication. Papers were excluded if they were not available in English. Full details of the inclusion/exclusion criteria are presented in Table 1.

**Table 1:** Inclusion and exclusion criteria.

Inclusion Criteria	Exclusion Criteria
**1) Female participants**	1) Articles that are not published in English or full text is not available
**2) Aged 18 years or older**	2) Studies including only DCIS or Stage IV patients
**3) Prescribed adjuvant hormone therapy for primary breast cancer**	3) Studies which do not include primary data such as systematic reviews and protocols
**4) Study conducted in either clinical practice or trials**	4) Studies investigating initiation to HT
**5) Studies present qualitative data on side effects of adjuvant hormone therapy**	5) Studies relating to screening or diagnosis
	6) Studies not using human subjects

### 2.3 Study selection

Search results were uploaded to ‘Covidence’ (Melbourne, Australia), an online tool used to manage systematic review screening and data extraction. After the removal of duplicates, titles and abstracts were independently screened by two authors (NP & SA) using a pre-defined screening tool based on the inclusion/exclusion criteria. If an abstract did not provide sufficient exclusion information, then the article was obtained for full text screening. Full text screening was conducted independently by the same two authors. Screening conflicts were resolved through discussion with a third reviewer (LF). Efforts were made to contact authors of papers where the full text was unavailable and to obtain full reports from conference abstracts.

### 2.4 Data extraction

Data were extracted from the method sections of included papers to obtain details of the study purpose, sample size, participant characteristics, study design and method of analysis. Data extraction was conducted by two authors (NP & SA) and all text labelled as ‘results’ or ‘findings’ were extracted to permit synthesis of all relevant data [22].

### 2.5 Quality appraisal

Selected studies were assessed for quality using the Joanna Briggs Institute Critical Appraisal Checklist for Qualitative Research [23]. Two authors (NP & SA) independently assessed the quality of the included studies by scoring each article against the checklist criteria of “met”, “not met”, “unclear” and “not applicable”. The quality criteria included domains related to method and methodology, data analysis, interpretation of results and reflexivity. An additional item was added to the quality assessment tool to assess whether the included studies had provided a clear description of adherence and persistence. This additional item was scored using the same checklist criteria. Any disagreements were resolved by consulting a third author (LF). In this review no study was eliminated based on quality assessment.

### 2.6 Data analysis

Data were analysed according to the thematic synthesis approach developed by Thomas & Harden [22]. This approach is influenced by both meta-ethnography and grounded theory [24]. The first stage of this approach is inductive, coding results line-by-line, then re-examining codes across studies to identify similarities. Following Thomas and Harden's [22] use of axial coding, codes were then re-assessed to ensure they accurately captured the data and additional levels of code were assigned when the text could be more appropriately represented. Descriptive themes were then developed by organising codes together into logical groups. The initial coding was conducted by SA, with NP contributing to the final interpretative stage. Analytical themes were developed from the data to explore women's lived experience of taking HT and the influence of HT side effects on adherence and persistence behaviour.

## 3 Results

### 3.1 Study characteristics

**Fig. 1:**
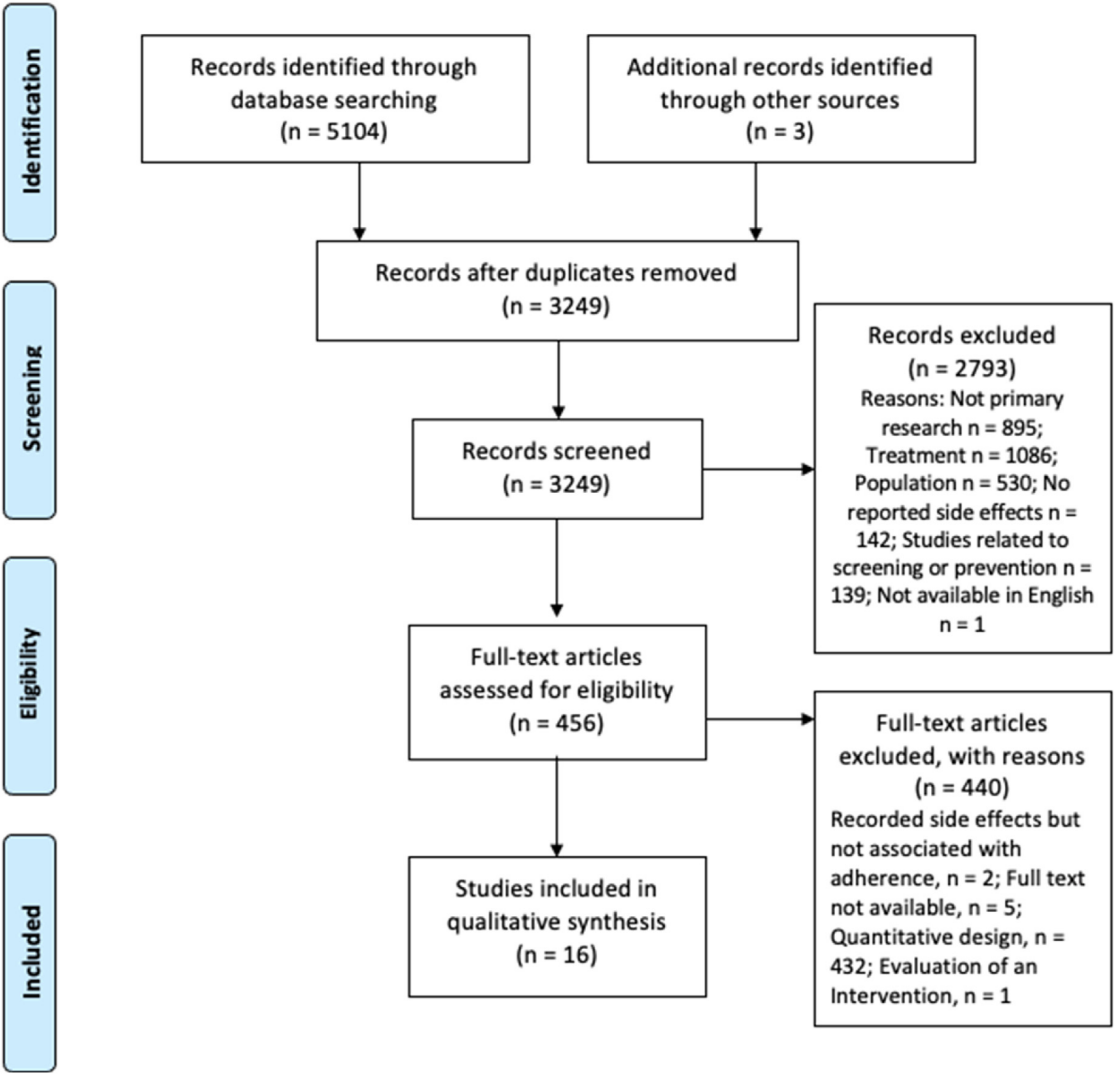
Prisma flow diagram of study selection.

A total of 5107 articles were identified. After removing quantitative studies, duplicates and screening titles and abstracts, 456 full-text articles were screened. Sixteen articles were included in the review. Full details of the search results and reasons for exclusion are shown in the PRISMA diagram (Fig. 1). The included studies were published between 2013 and 2019. Studies were published in 7 countries, with almost half (n = 7) originating from the USA. Sample sizes ranged from 12 to 54 participants: the total number of participants interviewed was 445 (excluding Mao et al. (2013) as this study included messages posted on a website) [25]. For full study characteristics information, see Table 2.

**Table 2:** Characteristics of included studies.

Study Reference	Country	Design/Method	Population	Sample Size	Sample Characteristics	Analysis	Research Question
Ahlstedt Karlsson et al. (2019) [33]	Sweden	Focus group	Women treated with Tamoxifen after breast cancer surgery	25	Median age: 62 years (range 42–80); 92% Married/co-habiting, 8% Single; 40% Retired; 60% Employed full or part time	Content analysis	To explore women's experiences with HT
Bedi (2018) [26]	USA	Focus group	Hormone receptor-positive breast cancer survivors under age 64 who had been prescribed ET since 2000; 50% on Tamoxifen, 41% on Aromatase Inhibitors, and 9% had switched.	22	Median age: 52 years when first prescribed ET (Range: 37–63 years); 64% Caucasian, 23% African American, and 14% Other Race; 18% High School Diploma, 14% Associate Degree, 41% Bachelors' Degree, 18% Post-Baccalaureate Degree, And 9% Preferred Not to Answer	Grounded theory	To understand from the survivor perspective which modifiable factors could have the greatest impact on the likelihood of HT continuation
Bluethmann et al. (2017) [34]	USA	Mixed method - interview	Breast cancer survivors who were prescribed adjuvant hormone therapy (i.e., Tamoxifen or AI).	27	Mean Age: 57 Years (Range = 49–86 Years); 27 Non-Hispanic White, 1 Non-Hispanic Black, 2 Hispanic; 22 Married, 8 Other; 4 High School Diploma, 5 Some College Or Technical School, 15 Bachelor's Degree, 6 Graduate Degree	Grounded theory	To describe survivors' reported appraisal and management of medication-related side effects and deconstruct survivors' decisions to initiate, discontinue, or maintain adjuvant hormone therapy.
Brauer et al. (2016) [38]	USA	Interview	Early-stage breast cancer, age 65 years and older taking AI	27	Mean age: 73.3 years (range 66–91); 4 Divorced, 1 Never married, 12 Married or living as married, 9 Widowed, 1 Separated;	Grounded theory	To explore how survivors of breast cancer made decisions about persisting with AIs, including specific challenges as well as attempts to manage them.
					7 High school diploma, 2 Some College, 9 Bachelor's Degree, 3 Some Graduate School, 6 Graduate Degree		
Brett et al. (2018) [27]	UK	Interview	Women who had been prescribed adjuvant hormone therapy, 19 Tamoxifen, 4 AI and 9 switched, 2–4 years following their diagnosis of breast cancer.	32	Adherers	Framework analysis	Identify the factors that influence whether women adhere to or do not adhere to adjuvant hormone therapy
					median age: 59 (Range 37–77)		
					15 (79%) Married, 1 (5%) Single, 3 (16%) Separated - Widow;		
					1 (5%) O′ O” level, GCSE, or equivalent, 16 (84%) College or university degree, 2 (11%) Postgraduate qualification;		
					Non-adherers		
					median age: 64 (Range 53–76);		
					10 (77%) Married, 2 (15%) Single, 1 (8%) Separated;		
					9 (69%) College or university degree, 4 (31%) Postgraduate qualification		
Cahir et al. (2015) [39]	Ireland	Interview	Women with stage I-III breast cancer prescribed adjuvant hormonal therapy purposively sampled by their medication taking behaviour at two cancer centres.	31	Mean age 51 years (SD ± 10);	Thematic analysis	To investigate influences on adjuvant hormonal therapy Medication Taking Behaviour in women with stage I–III breast cancer.
					7 Single, 24 Married/cohabiting;		
					Employed: 16 Yes, 15 No		
Cheng et al. (2017) [28]	China	Interview	Breast cancer survivors (<5 years after diagnosis)	19	Mean age: 54 years (range 41–65 years); 95% of the participants were unemployed or retired	Content analysis	Reveal Breast Cancer Survivor's views and experiences of self-management in extended survivorship
Harrow et al. (2014) [29]	UK	Interview	Women who had been prescribed tamoxifen or aromatase inhibitors (anastrozole or letrozole) and had been taking this medication for 1–5 years	30	Ages: <50 years 2 (7%), 50–64 years 15 (50%), ≥65 years 12 (40%), age unknown 1 (3%);	Constant comparison method applied within the framework approach	Women's experiences of taking adjuvant hormone therapy; their understandings and reasons for taking or not taking medication and the factors which influenced adherence or non-adherence and the information and support they received or desired.
					3 (10%) premenopausal, 7 (23%), perimenopausal, 17 (57%), postmenopausal, 3 (10%) unknown		
Humphries et al. (2018) [35]	Canada	Interviews and focus groups	Women aged 18 years or older, diagnosed with hormone receptor-positive breast cancer, had a first adjuvant hormone therapy prescription for early breast cancer within the last two years and sufficient fluency in French. 19 Tamoxifen, 3 Letrozole, and 21 Anastrozole	43	Ages: ≤49: 6, 50–59: 17, 60–69: 10, ≥70: 10;	Thematic Analysis	Identify women's attitudinal, normative, and control beliefs regarding adjuvant hormone therapy adherence that could be targeted by an intervention offered in the community pharmacy setting.
					2 Primary school, 8 Secondary school, 14 College, 19 University		
Iacorossi et al. (2018) [36]	Italy	Interview	Women aged between 44 and 75 years, diagnosed with breast cancer, who were being treated with endocrine therapy mainly Tamoxifen.	27	Median Age: 52 years, mean: 55.9 years, (range: 44–75 years);	Framework Analysis	To explore the experiences of adherence to hormone therapy in women with breast cancer.
					5 Single, 15 Married, 5 Divorced, 2 Widowed		
Lambert et al. (2018) [37]	Canada	Interview	Women diagnosed with HR + stage I to III breast cancer, without a prior cancer diagnosis, recurrence of breast cancer, or secondary cancer diagnosis (excluding non-melanoma skin cancer), who had completed primary cancer treatment, were fluent in English, aged 18–79 years at diagnosis, and prescribed Adjuvant Hormone Therapy.	22	Ages: 45–60 years 11 (50%), 60–79 years 11 (50%)	Thematic Analysis	To explore breast cancer survivors' experiences and perspectives of adjuvant hormone therapy use to describe how personal, social, and structural factors influence adjuvant hormone therapy persistence.
Mao et al. (2013) [25]	USA	Mixed method – online forum posts	Posts collected from breast cancer message boards between February 2002 and May 2010.	12 different breast cancer message boards	Data on sample not collected.	Content Analysis	To evaluate the volume and frequency of AI-associated side effects reported on internet message boards.
Moon et al. (2017) [30]	UK	Interview	Breast cancer survivors who had been prescribed tamoxifen	32	Mean age: 55 years (range: 36 to 77, SD = 10.6); 16 Post-Menopausal, 5 Pre-Menopausal, 5 Perimenopausal, 6 Unsure; 24 White, 5 Black British, 2 Asian/Asian British, 1 Mixed	Thematic Analysis	To elicit abroad understanding of women's lived experiences of tamoxifen, their motivation to adhere to treatment and identify reasons for non-adherence and non-persistence, in their own words.
Pieters et al. (2019) [40]	USA	Interview	Women aged at least 65 years old, started an AI for loco-regional (Stage I, II or III) breast cancer 4–36 months prior to enrolment and were in charge of taking their own medications.	54	Mean age: 73.3 years (range = 66–91);	Thematic Analysis	To describe and compare how women treated for primary early-stage breast cancer either persisting or not persisting with an AI received, interpreted, and acted upon AI-related information.
					44 White, 3 Latina, 3 Japanese, 2 Chinese, 1 Korean, 1 African American; 24 Married, 2 Never married, 16 Widowed, 10 Divorced, 2 Separated;		
					9 High School Graduate, 7 Some College, 19 College Graduate, 5 Some Graduate School, 14 Graduated Grad School; Household Income: 14: $21k-$40,999, 8: $41k-60,999, 13: $61k-80,999, 5: $81k-100,999, 12: >$101k		
Van Londen et al. (2014) [31]	USA	Focus group	Breast cancer survivors, aged 50 years or older, with Adjuvant Hormone Therapy-related symptoms	14	Mean age: 58.8 years (SD: 6.7); 100% Caucasian; 79% Married; 21% Employed, 79% Not Working (retired, disabled, not able to find a job)	Thematic Analysis	To explore survivors' recollection of the conversation with the medical oncologist about starting Adjuvant Hormone Therapy, experiences with Adjuvant Hormone Therapy-related symptoms, Adjuvant Hormone Therapy-related symptom management, challenges to taking Adjuvant Hormone Therapy, and views about how Adjuvant Hormone Therapy-related symptoms might be better managed.
Wells et al. (2016) [32]	USA	Interview	Underserved breast cancer survivors at a comprehensive cancer centre in the south eastern United States.	25	Mean age: 59.92 (Range 46–71 years; SD = 6.82);	Content Analysis	To evaluate the barriers and facilitators to taking anti-hormonal medications among medically and historically underserved breast cancer survivors within the first five years post chemotherapy, radiation, and/or definitive surgery.
					(15 = no, 10 = yes) Hispanic/Latina, 13 White, 7 African American, 1 Asian, 4 other; 4 Single, 8 Married, 11 Separated, 2 Widowed;		
					11 Not Currently Employed, 8 Part-Time, 6 Full-Time		

Side effects were self-reported by participants and both physical and psychological side effects from HT were reported in the included studies. The most commonly reported physical side effects were fatigue [[26], [27], [28], [29], [30], [31], [32], [33]], hot flashes [26,27,[29], [30], [31],[33], [34], [35], [36], [37]], night sweats [29,31,33,34,37], musculoskeletal pain [[25], [26], [27],^29^,[31], [32], [33], [34], [35], [36], [37], [38]], weight gain [[26], [27], [28], [29],^32^,^33^,^37^], alopecia [29,32,33,35,37], sexual dysfunctions including loss of sexual interest [27,29,30,33,36], vaginal dryness [26,27,29,31,[33], [34], [35], [36], [37]] and pain during sex [26,33]. The most commonly reported psychological side effects included depression [26,27,[30], [31], [32],^35^,^36^,^38^], insomnia or sleep disturbance [[32], [33], [34], [35],^40^], suicidal feelings [29], mood swings [27], impaired memory and concentration [25,30,31,34], anxiety [[30], [31], [32]] and anger [26,33]. Two studies [39,40] discussed side effects generally, and did not specify which side effects were experienced by participants. Details of specific side effects reported in each study are included in Table 3.

**Table 3:** Reported side effects.

Side Effect	N	Study Side Effect Reported in
**Alopecia**	5	Ahlstedt Karlsson et al. (2017), Harrow et al. (2014), Lambert et al. (2018), Wells et al. (2016), Humphries et al. (2018)
**Anxiety**	3	Moon et al. (2017), Van Londen et al. (2014), Wells et al. (2016)
**Brittle Fingernails**	1	Bleuthmann et al. (2017)
**Cognitive dysfunction (memory problems, issues with concentration)**	4	Bleuthmann et al. (2017), Mao et al. (2013), Van Londen et al. (2014), Moon et al. (2017)
**Constipation**	1	Wells et al. (2016)
**Diarrhoea**	1	Lambert et al. (2018)
**Depression/low mood/mood swings**	11	Ahlstedt Karlsson et al. (2017), Bedi et al. (2019), Braeur et al. (2016), Brett et al., 2018), Humphries et al. (2018), Iacorossi et al. (2018), Moon et al. (2017), Wells et al. (2016), Brett et al. (2018), Van Londen et al. (2014), Wells et al. (2016)
**Fatigue**	8	Ahlstedt Karlsson et al. (2017), Cheng et al. (2017), Harrow et al. (2014), Moon et al. (2017), Van Londen et al. (2014), Wells et al. (2016), Brett et al., 2018), Bedi et al. (2019),
**Facial Flushing**	1	Bleuthmann et al. (2017)
**Hot Flashes**	12	Ahlstedt Karlsson et al. (2017), Bedi et al. (2019), Bleuthmann et al. (2017), Humphries et al. (2018), Iacorossi et al. (2018), Lambert et al. (2018), Van Londen et al. (2014), Van Londen et al. (2014), Wells et al. (2016), Brett et al. (2018), Harrow et al. (2014), Moon et al. (2017),
**Insomnia/sleep disturbances**	5	Harrow et al. (2014), Moon et al. (2017), Van Londen et al. (2014), Wells et al. (2016) Lambert et al. (2018)
**Loss of Sexual interest**	5	Ahlstedt Karlsson et al. (2017), Brett et al. (2018), Iacorossi et al. (2018), Harrow et al. (2014), Moon et al. (2017),
**Liver Problems**	1	Iacorossi et al. (2018)
**Musculoskeletal pain**	12	Ahlstedt Karlsson et al. (2017), Bedi et al. (2019), Bleuthmann et al. (2017), Braeur et al. (2016), Harrow et al. (2014), Humphries et al. (2018), Iacorossi et al. (2018), Lambert et al. (2018), Mao et al. (2013), Van Londen et al. (2014), Wells et al. (2016), Brett et al., 2018)
**Nausea**	2	Harrow et al. (2014), Wells et al. (2016),
**Osteoporosis**	1	Cheng et al. (2017)
**Pain during sex**	2	Ahlstedt Karlsson et al. (2017), Bedi et al. (2019),
**Side effects not specified**	2	Pieters et al. (2019), Cahir et al. (2015)
**Suicidal feelings**	1	Harrow et al. (2014)
**Sexual Dysfunction**	1	Van Londen et al. (2014)
**Sweating/night sweats**	5	Ahlstedt Karlsson et al. (2017), Bleuthmann et al. (2017), Harrow et al. (2014), Lambert et al. (2018), Van Londen et al. (2014),
**Vaginal dryness**	9	Ahlstedt Karlsson et al. (2017), Bedi et al. (2019), Bleuthmann et al. (2017), Brett et al. (2018), Harrow et al. (2014), Humphries et al. (2018), Iacorossi et al. (2018), Lambert et al. (2018), Van Londen et al. (2014)
**Weight gain**	7	Ahlstedt Karlsson et al. (2017), Bedi et al. (2019), Brett et al., 2018), Cheng et al. (2017), Harrow et al. (2014), Lambert et al. (2018), Wells et al. (2016)

### 3.2 Quality assessment

Overall, the methodological quality of the included studies was judged to be high. However, only 50% of studies provided a clear description of adherence and persistence. Details of the quality assessment can be seen in Table 4.

**Table 4:** Results of the quality assessment of included studies.

Study Reference	Q1	Q2	Q3	Q4	Q5	Q6	Q7	Q8	Q9	Q10	Q11^a^
Ahlstedt Karlsson et al. (2019)	Y	Y	Y	Y	Y	N	N	Y	Y	Y	N
Bedi (2019)	Y	Y	Y	Y	Y	N	N	Y	Y	Y	N
Bluethmann et al. (2017)	Y	Y	Y	Y	Y	N	N	Y	Y	Y	N
Brauer et al. (2016)	Y	Y	Y	Y	Y	Y	N	Y	Y	Y	N
Brett et al. (2018)	Y	Y	Y	Y	Y	N	N	Y	Y	Y	Y
Cahir et al. (2015)	Y	Y	Y	Y	Y	N	N	Y	Y	Y	Y
Cheng et al. (2017)	Y	Y	Y	Y	Y	Y	N	Y	Y	Y	N
Harrow et al. (2014)	Y	Y	Y	Y	Y	Y	N	Y	N	Y	Y
Humphries et al. (2018)	Y	Y	Y	Y	Y	N	N	Y	Y	Y	Y
Iacorossi et al. (2018)	Y	Y	Y	Y	Y	N	N	Y	Y	Y	Y
Lambert et al. (2018)	Y	Y	Y	Y	Y	N	N	Y	Y	Y	Y
Mao et al. (2013)	Y	Y	Y	Y	Y	N	N	N	Y	Y	N
Moon et al. (2017)	Y	Y	Y	Y	Y	N	N	Y	Y	Y	Y
Pieters et al. (2019)	Y	Y	Y	Y	Y	Y	Y	Y	Y	Y	Y
Van Londen et al. (2014)	Y	Y	Y	Y	Y	N	N	Y	Y	Y	N
Wells et al. (2016)	Y	Y	Y	Y	Y	Y	N	Y	Y	Y	N

### 3.3 Thematic synthesis

Four analytical themes were derived from the data. The first, *‘Daily impact of HT side effects’*, relates to the influence of HT on all aspects of functioning, including work, relationships, social life and physical and mental health. The second theme, ‘*Role of Health Care Professionals (HCPs)*’, highlights the important role that HCPs play in a patient's HT adherence behaviour. Third, *‘Managing HT side effects’* summarises the strategies used by patients to reduce HT side effects. Finally, *‘Weighing up the pros and cons’* highlights the key aspects involved in HT adherence and persistence decision making. For each of these analytical themes, several descriptive themes were identified, which are detailed in Table 5 below.

**Table 5:** Analytical themes, descriptive themes and illustrative extracts.

Analytical theme	Descriptive themes	Illustrative extracts
Daily impact of HT side effects	Social functioning	“I started to withdraw from social situations. I didn't trust my body to co-operate. I missed out on quite a few things, because I was too afraid that [due to the diarrhoea] I would have to run or, change my clothes or have a shower. And make a mess in public. Emotionally, it was devastating” **(Lambert et al., 2018; p.5)** “It just stops you getting on with your life. You have been through surgery, then chemotherapy, then you take the hormone drugs. You get to the stage when you want to get back to normal, but these drugs stop you doing that” **(Brett et al., 2018; p.296)**
	Inter-personal relationships	“One of the things that upset me most at the time [was that] I lost all interest in sex overnight – it didn't help my husband as you can imagine.” **(Brett et al., 2018; p.294)** “And I have two, three grandchildren. I love children …. So, when I see them, I want to play with them … but physically I can't do it. So, that makes me—really upsets me. I think that's the thing.” **(Brauer et al., 2016; p.995)**“Well your friends and relatives don't want to hear about it [symptoms].” **(Van Londen et al., 2014; p.5)**
	Ability to work	“I am more forgetful. I work harder at work to do the same job that I used to just do. It's harder for me to stay focused, to concentrate, to think clearly, to remember everything.” **(van Londen et al., 2014; p.5)** “I am unable to undertake too heavy/many physical tasks. I should perform light work only. For example, I easily feel tired when cooking. I have to take a break and lie down on the bed for 15 min. After boosting my energy, I get up and continue to cook.” **(Cheng et al., 2017; p.1043)**
	Physical health	“There are days that all of you is in pain, all the body …. A pain that you don't know what is hurting …. and it is so horrible … you try to be still so it doesn't hurt. You can't cook, you can't clean, you can't even bathe because … the pain is in all your body.” **(Wells et al., 2016; p.7)** “I felt like a 90-year-old woman.” **(Bluethmann et al., 2017; p.6)**
	Mental Wellbeing	“I just don't feel exactly like myself [on Arimidex®]. I don't feel real clear-headed, and I feel groggy a lot of the time. If you're not sleeping well, you don't know if one thing causes the other.” **(Bluethmann et al., 2017; p.6)** “I felt so low, was having suicidal thoughts, really didn't feel like myself at all, I was in so much pain and that I'd made the decision that I was going to come off tamoxifen.” **(Moon et al., 2017; p.18)**
Role of Health Care Professionals	Unprepared for side effects	“I didn't even know my body was going to go through that. It hit me like a boom” **(Bluethmann et al., 2017; p.5)** “My doctor told me I would probably have night sweats and hot flashes, but that's all I really expected. I didn't expect the [severe side effects] I had. … It started with pain in my shoulders, and then it moved to my jaw. Eventually, it moved to every joint in my body.” **(Bluethmann et al., 2017; p.5)** “I would have liked more information to prepare for the side effects. I was given lots of information about the side effects of chemotherapy and how to manage them, but I wasn't expecting the side effects of HT. So perhaps that made it worse” **(Brett et al., 2018; p.293)**
	Feeling unsupported by HCP	“You feel like you don't have all the structures we talked about before [when undergoing chem/radiotherapy], so now [on hormone therapy]you're winging it, and that's scary” **(Braeur et al., 2016; p.993)** “This is the one thing that I do find a lot of women struggling most with, that they feel so … they're just not listened to. They're not being validated in what they're experiencing.” **(Moon et al., 2017; p.22)** “I would rather have somebody tell me that they don't know why I had a reaction to this or that, rather than just make me feel like I'm a child or it's just your hormones or it's just your mental incapacity” **(van Londen et al., 2014; p.6)** “I wanted to be followed up. If they're going to start fiddling with your hormone levels, they should be checking you every three months. There's no checks and balances. If I had felt I was being followed and people knew what was happening to me, I would have felt much better. I felt totally alone” **(Lambert et al., 2018; p.8)**
	HCP's influence on adherence	“I said ‘I've decided to stop [taking hormone therapy], what do you think?’ And she [oncologist] shrugs and said ‘Fine’. If she had said ‘No, definitely not, I really don't think you should stop’, I probably wouldn't have” **(Lambert et al., 2018; p.7)** I had a long conversation with him [GP] about it – he really cared. He swapped me on to this one – I know he is doing what he can. If you feel someone cares, it kind of encourages you to keep going, if you know what I mean. [P18 adherer] **(Brett et al., 2018; p.293)**
Managing HT side effects	Non-adherence in order to reduce side effects	“I was walking with crutches and canes for support, but after I stopped taking the tamoxifen, the pain subsided.” **(Bluethmann et al., 2017; p.7)** “… the hot flashes. I would wake up during the night and be drenched. I skipped one month [of ADJUVANT HORMONE THERAPY].” **(Humphries et al., 2018; p.8)**
	Self-help strategies	“I read on the internet about looking after myself […] I eat much healthier now, and avoid alcohol and caffeine. A bit boring, but it helps. The side effects are less now” **(Brett et al., 2018; p.293)** “Very often I get home (from an appointment) and I realize that I don't have the full picture, and so then I go to the Internet.” **(Pieters et al., 2019; p.9)** “I mentioned it to [my doctor], but I knew it was just one of those things I would have to cope with, so I just did.” **(Bluethmann et al., 2017; p.6)**
	Social support	“I realize, hey, I'm not the only one that's going through this. Other people are going through this too … We're a community.” (**Pieters et al., 2019; p.9)** “You can be totally blunt with him. And he just really wants to help do what's best for you, but he listens to your issues. He doesn't minimize how you're feeling” **(Bedi, 2018; p.80)**
Weighing up the pros and cons	Accepting risk of cancer recurrence	“I've had tamoxifen, and I've had breast cancer. I would rather have breast cancer.” **(Bluethmann et al., 2017; p.7)”** “You're counting the days and it becomes like you can't wait for the end [of ADJUVANT HORMONE THERAPY]. I don't know what's going to happen. It may come back and I'm going to die anyway. So, I'd rather have a good quality of life while I'm alive and not have side effects” **(Lambert et al., 2018; p.8)** “I called my doctor and told her I was going to stop taking Femara, that it was affecting me adversely, and that my quality of life was more important.” **(Bluethmann et al., 2017; p.6)**
	Fear of cancer recurrence	“The advantage they told me, was that it could save me. […] I saw this as prevention against a recurrence.” **(Humphries et al., 2014; p.6)** “If it was for anything else other than the cancer I would have stopped it, there's no questions, but because the cancer is such a big thing, you know the possible return of it, that's the only reason I'm struggling with it” **(Moon et al., 2017; p.20)** “I will carry on despite how I feel just because it's the only thing I can do and I'll do anything I can [to stop the cancer returning] because of the kids really – I do want to be around [for them” **(Brett et al., 2018; p.294)**

#### 3.3.1 Daily impact of HT side effects

Many women reported experiencing intense side effects from HT, describing them as “violent” and “excruciating” [37]. Side effects were reported to significantly impact on women's quality of life, which as a result impacted upon their mental wellbeing. This created a heavy emotional burden which led women to question their identity and to experience depressive and suicidal states [30,32,33,36,38]. Physical side effects such as joint pain had a debilitating effect, leaving one individual reliant on a cane for walking [32]. Several participants experienced pain throughout the whole body, severely restricting their movement. This limited their ability to perform household chores, personal care and even simple tasks like getting out of bed in the morning [30,32]. Ability to perform physical tasks at work was also affected [31]. As well as restricting physical capability at work, cognitive side effects of hormone therapy (including difficulty concentrating and memory loss) made it difficult for women to focus on tasks [41]. Women also described themselves as more forgetful, which was exacerbated by the interruption of sleep due to hot flushes, night sweats and joint pain [31,34,35,37]. This loss of sleep was related to lack of energy for social activities and professional responsibilities [31]. Professional confidence was also affected by body image concerns due to weight gain [38]. In addition to debilitating physical side effects, the “disabling” effect [36] of adjuvant HT treatment had severe emotional implications. Women became socially withdrawn and avoided typical daily activities due to fear of embarrassment from unpredictable side effects and losing trust in their own bodies [37]. A loss of sexual desire and menopausal symptoms such as hot flashes and vaginal dryness put strain on romantic relationships [33,38], which was distressing for several participants [36,38]. Women also reported that side effects impacted on other interpersonal relationships, such as relationships with family members and friends, as they felt that they were unable to interact with them as they had done previously [38], or that they could not talk to them about the side effects they were experiencing [31]. Menopausal symptoms also impacted women's sense of identity, as symptoms such as musculoskeletal pain, loose teeth, vaginal dryness and alopecia created a sense of premature ageing, leading one individual to describe feeling “like a 90-year-old woman” [34].

#### 3.3.2 Role of Health Care Professionals (HCPs)

A lack of pre-emptive information about the nature and severity of HT side effects was highlighted consistently in the literature, with women reporting feeling unprepared for what they experienced. Participants explained that they had been informed of the side effects of primary treatment such as chemotherapy [27], but expressed the desire to be forewarned about the potential side effects of hormone treatment [26]. Some had been prepared for side effects but were surprised by the range and intensity of the menopausal symptoms [34]. Women stated that this lack of information was a significant issue, as informed expectations would have helped them to cope better and potentially, to adhere more closely to their prescription [27,30]. Many struggled to identify whether menopausal symptoms were due to HT, normal ageing or pre-existing conditions [38] and this confusion was linked to delaying seeking help, which led to worsening of side effects and in some cases, discontinuation of treatment [40]. Although some women recalled having initial conversations about the purpose of adjuvant treatment, they struggled to describe these conversations, and many were sure they had received little warning about potential side effects [31]. Many were informed of adjuvant treatment during their first cancer treatment consultation and had been too “bombarded” [27] with information to fully comprehend or remember what information they had been given.

The transition from “receiving” primary treatment (chemotherapy, surgery and radiotherapy) [38] to being largely responsible for their own medication use, marked a significant change for breast cancer survivors. Participants described “falling through the gaps” [27] of the healthcare system after finishing primary treatment, with some feeling that their primary care practitioner did not possess sufficient specialist knowledge to support them with adjuvant HT [26,33] and that other Health Care Professionals (HCPs) (such as breast cancer nurses and oncologists) [29,31] were too busy supporting patients in primary therapy to answer their questions. This resulted in women feeling unsupported and left to manage side effects on their own [37]. Women who did discuss side effects with their HCPs were often not satisfied with the outcome of the consultation. They expressed a desire for more personalised advice, describing the guidance they received as “repetitive and too general” [26]. Some described having their concerns minimised and perceived HCPs as uncaring about their suffering [30,31]. Women stated there is a need for more psychological support with side effects [30,36,37], and more monitoring of side effects to support them in taking the medication. One individual stated that more encouragement from her HCP to persist with medication would have made her reconsider her decision to discontinue [37]. Having trust in HCP's good intentions and expertise was linked to willingness to follow their advice [26,27,35], therefore, a supportive relationship with HCPs could potentially improve adherence to medication. This was highlighted by one participant explaining they discontinued adjuvant treatment without consulting a doctor because they felt “nobody was interested” [38].

#### 3.3.3 Managing HT side effects

Many women were proactive in their self-management of side effects, conducting their own research (largely online) to deepen their understanding of their medication and identify ways to reduce side effects. Strategies included: dietary changes, increased exercise, wearing thinner clothes, taking breaks during household chores, and minimising activity in swollen limbs [27,28,38]. This online research led some to reconsider their decision to continue taking HT [40], as they realised their symptoms were caused by their medication.

Online breast cancer survivor communities were commonly utilised as a source of information [26,29,40]. Women valued experiential support from others who had taken HT and could provide support on how to manage side effects [26]. These communities were also a significant source of social and emotional support, as women felt a sense of relief upon realising, “I'm not the only one that's going through this” [40]. Women also valued the social support they received from their personal networks, who helped to support them with side effects and encouraged persistence when their motivation waned [37]. HCPs could play a particular role in this, as HCPs who listened to women's experiences and did not minimize their side effects [26], were considered more supportive and encouraging of adherence and persistence.

Some participants made considered decisions not to adhere to their prescriptions or to discontinue their prescriptions completely to get relief from the side effects of HT [38]. Participants took “medication holidays” to alleviate side effects including hot flushes, night sweats (disrupting sleep), joint pain and depressive symptoms, and nausea [29,34,35]. One study [29] stated that women who were advised to take medication breaks by their HCPs were more likely to resume their treatment, compared to those women who took medication breaks on their own initiative. This highlights the need for HCPs to actively support women experiencing side effects in order to facilitate HT adherence.

#### 3.3.4 Weighing up the pros and cons

The influence of side effects on quality of life led to many women reconsidering their decision to persist with treatment. This decision was influenced by many factors including side effect severity, fear of cancer recurrence and perceived risk of recurrence. Side effect severity appears to influence an individual's decision of whether to persist or discontinue HT. Some women coped relatively well with HT side effects [29], whereas some described the treatment as “potentially worse than the disease” and “unbearable” [37]. The intensity of side effects was a key factor in the decision to discontinue therapy, as many reached a stage where they felt the advantages of the drugs were outweighed by the burden of side effects [31,33,34,37]. This was prominent in older women, who explained they would prioritise quality of life in their remaining years over minimising the risk of recurrence due to wanting to “enjoy the life you have left”. Similarly, many expressed a desire to move on from cancer treatment and resume normality, which they felt HT was inhibiting [40].

The advantages of HT were well-understood by the majority of breast cancer survivors. Adjuvant treatment was considered a “security blanket” [37], giving women hope for remaining cancer-free in the future [36]. This was a powerful motivator, as women expressed a constant fear of cancer recurrence, characterising this worry as, “a sword dangling above your head” [30]. Awareness of potential recurrence was a factor in anticipated regret. Women expressed wanting to know that they had “done all the right things” [36] to prevent recurrence. A recurring sentiment among survivors was that adjuvant treatment was not optional, and they were obliged to persist due to family responsibilities [27,36] and a duty to the people who had supported them through treatment [35]. Managing the side effects from HT was described as a “small price to pay” [30] for the potential benefits.

Although HT gave some a sense of security against recurrence, this was often dependent on how much women perceived themselves to be at risk. Some women were confident in their decision to discontinue or persist due to specific characteristics (age, cancer sensitivity, and cancer hormone receptor status) [35], whereas some felt oncologists had not been able to give them a clear indicator of their risk of recurrence [27]. Some were sceptical of the benefits of HT due to the lack of tangible efficacy, and confusion regarding its influence on risk of recurrence [37]. This demonstrates an opportunity for HCPs to facilitate adherence, by reminding women of the purpose of HT treatment, normalising their experience of side effects [35], and encouraging them to persist [27].

## 4 Discussion

This qualitative review aimed to identify how side effects of hormone therapy (HT) can influence breast cancer survivors adherence to and persistence with their medication. Identifying the impact of side effects on adherence to and persistence with HT for breast cancer survivors is critical for reducing risk of recurrence and reducing mortality. Four analytical themes were identified as having a significant influence on HT adherence and persistence behaviour. These themes were: “*Daily impact of HT side effects*”, “*Role of Health Care professionals (HCPs)*”, “*Managing HT Side effects*”, and “*Weighing up the pros and cons*”. These findings are consistent with the results of Clancy et al. (2020) [20] and Lambert et al. (2018) (19), who both identified that side effects had a considerable impact on quality of life and consequently adherence and persistence, and that there is a need for more follow-up care and support from health care professionals. The current review progresses previous reviews through its detailed examination of the specific relationship between side effects and adherence and persistence with HT, whereas earlier reviews of the qualitative literature simply aimed to identify studies which examined adherence and persistence.

There is strong evidence that the presence and severity of side effects can impact on adherence and persistence behaviour [41]. The current review revealed that HT side effects had wide-ranging impacts on breast cancer survivors' daily life. Musculoskeletal pain limited mobility and impacted on sleep, which then impacted cognitive functioning (i.e. concentration and memory), thus reducing women's ability to work. HT side effects also affected women's ability to socialise and enjoy fulfilling sexual relationships. Symptom-specific interventions have been trialled, with some evidence suggesting that use of medications can reduce the severity of side effects [42]. However, some side effects (i.e. sleep disturbance, fatigue) cannot be resolved through medical interventions, as pharmacological treatments are not recommended for long term use [43]. Therefore, psychological interventions such as mindfulness and/or cognitive behavioural therapy, and lifestyle interventions such as physical activity, which are effective at improving sleep and fatigue in a variety of clinical populations [44,45], also need to be trialled with breast cancer survivors to assess whether reducing these side effects promotes HT adherence and persistence.

The impact of side effects can result in woman choosing between a reduction in cancer risk and maintaining their quality of life. This evaluation process appears to be pivotal to the decision to adhere and persist with adjuvant hormone therapy, as survivors appear to actively decide if the benefits of treatment outweigh the detrimental impact side effects have on their quality of life [33,34]. The findings of this review also emphasised how women often felt unprepared for the side effects of HT. If women are to persist with HT, then they require adequate information about the benefits of treatment and the risk of recurrence that is tailored to their needs [46]. Previous research has suggested that informing women with breast cancer about HT and potential side effects can be a successful strategy to enhance adherence [47]. However, providing information alone is not sufficient for improving adherence and persistence [48]. Adequate health care provider support and communication needs to be provided to support long term adherence and persistence.

The results of this review also highlighted the significance of the important role healthcare professionals play in supporting women to manage HT side effects and encouraging adherence and persistence behaviour. This review has identified that breast cancer patients who receive HT often receive this treatment whilst in a transitional period from primary to secondary care, and a result patients often feel they are no longer a priority or find that they are not able to receive the specialist care that they require. This lack of support indicates a missed opportunity for early intervention and promotion of the benefits of persisting with adjuvant HT, as some woman reported that more encouragement and support from healthcare professionals would help them to cope better with their side effects [19].

A lack of support from healthcare providers leads to survivors developing their own self-management strategies for dealing with medication side effects, which in some cases results in non-adherence [29,34]. Patient-centred communication is a positive predictor of adherence [49], with evidence suggesting that better communication improves patient outcomes and reduces the burden of symptoms [50]. Communication with healthcare providers and individually tailored approaches are therefore needed for effective treatment planning [19]. Comprehensive follow-up care should be provided for women who are prescribed adjuvant HT so that those experiencing adverse effects can be identified and supported to continue with their medication as prescribed.

Studies included within this review tended to combine all types of HT rather than consider any differences between different treatment approaches. Clinical observations suggest that different treatments (i.e. Tamoxifen and AI) have different side effect profiles, albeit with some overlap. This review was not able to disentangle if there were any differences between the impact of side effects based on treatment type, and whether this had any impact on adherence and persistence. This highlights a limitation of the literature, as evidence indicates that side effect burden impacts on the decision to persist with treatment. Therefore, being able to identify if one treatment option is less impactful may help to promote adherence and persistence, and would be clinically meaningful for both patients and practitioners.

A limitation of this review is that it focuses specifically on women with breast cancer and their experience of HT side effects. Approximately 350 men are diagnosed with breast cancer annually in the UK [51], and whilst this is considerably less than the number of women, it is still an important issue that should be considered in future research. Men with breast cancer can also be treated with HT such as tamoxifen, however they may experience different side effects. However, there is a lack of research on men with breast cancers experience of taking HT and they may experience different side effects or have other barriers to adherence. Another limitation of this review was that it was not possible to determine the impact of prior treatment on adherence and persistence, or the impact of age or ethnicity, which may impact the side effects experienced. Due to the qualitative nature of the included studies and the lack of distinctions made in the original studies it is difficult to draw any conclusions. However, the authors are currently completing a review that focuses on the quantitative literature related to this topic and are hoping to unpick the impact of prior treatment, age and ethnicity differences further in that review.

## 5 Conclusion

This paper aimed to explore how side effects influence adherence and persistence to HT. This review has identified key areas for developing interventions to improve HT adherence and persistence in clinical practice. By identifying the precise ways in which side effects contribute to non-adherence and treatment discontinuation, interventions can be offered which reduce the impact of side effects and remove some of the barriers to breast cancer survivors taking their medication as prescribed.

## Declaration of competing interest

There are no competing interests to declare.

## Data Availability

The protocol for this review is available on PROSPERO (CRD42020192481).
