# Supplementary material for: The impact of medication side effects on adherence and persistence to hormone therapy in breast cancer survivors: A qualitative systematic review and thematic synthesis
Source: Breast. 2021 May 17;58:147–59. doi: 10.1016/j.breast.2021.05.005 (PMC8165559; doi:10.1016/j.breast.2021.05.005)
Supplement: Multimedia component 1 [file mmc1.docx]

# Search Strategy

| Patient Population | Interest | Context |
| --- | --- | --- |
| Breast cancer patients on adjuvant endocrine/ hormonal therapy | Adherence | Impact of side effects |
| breast cancer*  breast neoplasm*  Adjuvant breast cancer  Breast cancer survivor  Tamoxifen  Anastrozole  Arimidex  Exemestane  Aromasin  Letrozole  Femara  Hormonal therapy  Aromatase inhibitor* hormon* therapy  Endocrine therapy*  Selective estrogen receptor modulators  SERM  Adjunctive Treatment Adjuvant Treatment | Medication adherence  Patient Compliance  adher*  complian*  persist*  discont* | Adverse effects  Side effects  Vasomotor  Hot flushes  Musculoskeletal  pain  Vulvovaginal  menopausal  gynaecological  weight  weight gain  social function  Quality of life  cognitive dysfunction  fatigue  Sleep*  sleep problems  insomnia  depression  anxiety  mental wellbeing |

| Database | Dates | Search Terms |
| --- | --- | --- |
| Medline | 1946 – 13^th^ March 2020 | 1. Breast cancer.mp. or Breast Neoplasms/ 2. Adjuvant breast cancer.mp. 3. Cancer Survivors/ or Breast cancer survivor.mp. 4. 1 or 2 or 3 5. Patient Compliance/ or Medication Adherence/ 6. (adher* or complian* or persist* or discont*).mp. [mp=title, abstract, original title, name of substance word, subject heading word, floating sub-heading word, keyword heading word, organism supplementary concept word, protocol supplementary concept word, rare disease supplementary concept word, unique identifier, synonyms] 7. 5 or 6 8. Tamoxifen/ae [Adverse Effects] 9. Tamoxifen.mp. 10. Anastrozole/ae [Adverse Effects] 11. Anastrozole.mp. 12. Arimidex.mp. or Anastrozole/ 13. Exemestane.mp. 14. Aromasin.mp. 15. Letrozole/ae [Adverse Effects] 16. Femara.mp. 17. Hormonal therapy.mp. 18. Aromatase Inhibitors/ or Aromatase inhibitor*.mp. or Aromatase/ 19. Endocrine therapy.mp. 20. Selective estrogen receptor modulators.mp. or Selective Estrogen Receptor Modulators/ 21. SERM.mp. 22. Adjunctive Treatment.mp. 23. Adjuvant Treatment.mp. 24. 8 or 9 or 10 or 11 or 12 or 13 or 14 or 15 or 16 or 17 or 18 or 19 or 20 or 21 or 22 or 23 25. Adverse effects.mp. 26. Side effects.mp. 27. Menopause/ or Hot Flashes/ or Vasomotor System/ 28. Musculoskeletal.mp. or Musculoskeletal Pain/ 29. Cancer Pain/ or Low Back Pain/ or Musculoskeletal Pain/ or Pain/ 30. Vulvovaginal.mp. 31. gynaecological.mp. 32. weight.mp. 33. Weight gain.mp. or Weight Gain/ 34. "Quality of Life"/ 35. Social function.mp. 36. Cognitive dysfunction.mp. or Cognitive Dysfunction/ 37. fatigue.mp. or Fatigue/ 38. Sleep problems.mp. or Sleep/ 39. insomnia.mp. 40. Depression/ 41. Anxiety/ 42. Mental wellbeing.mp. or Mental Health/ 43. 25 or 26 or 27 or 28 or 29 or 30 or 31 or 32 or 33 or 34 or 35 or 36 or 37 or 38 or 39 or 40 or 41 or 42 44. 4 and 7 and 24 and 43 45. limit 44 to English language |
| PsycINFO |  |  |
| Cochrane Library |  |  |
| Embase | 1947 – 13^th^ March 2020 | 1. Breast cancer.mp. or Breast Neoplasms/ 2. Adjuvant breast cancer.mp. 3. Cancer Survivors/ or Breast cancer survivor.mp. 4. 1 or 2 or 3 5. Patient Compliance/ or Medication Adherence/ 6. (adher* or complian* or persist* or discont*).mp. [mp=title, abstract, original title, name of substance word, subject heading word, floating sub-heading word, keyword heading word, organism supplementary concept word, protocol supplementary concept word, rare disease supplementary concept word, unique identifier, synonyms] 7. 5 or 6 8. Tamoxifen/ae [Adverse Effects] 9. Tamoxifen.mp. 10. Anastrozole/ae [Adverse Effects] 11. Anastrozole.mp. 12. Arimidex.mp. or Anastrozole/ 13. Exemestane.mp. 14. Aromasin.mp. 15. Letrozole/ae [Adverse Effects] 16. Femara.mp. 17. Hormonal therapy.mp. 18. Aromatase Inhibitors/ or Aromatase inhibitor*.mp. or Aromatase/ 19. Endocrine therapy.mp. 20. Selective estrogen receptor modulators.mp. or Selective Estrogen Receptor Modulators/ 21. SERM.mp. 22. Adjunctive Treatment.mp. 23. Adjuvant Treatment.mp. 24. 8 or 9 or 10 or 11 or 12 or 13 or 14 or 15 or 16 or 17 or 18 or 19 or 20 or 21 or 22 or 23 25. Adverse effects.mp. 26. Side effects.mp. 27. Menopause/ or Hot Flashes/ or Vasomotor System/ 28. Musculoskeletal.mp. or Musculoskeletal Pain/ 29. Cancer Pain/ or Low Back Pain/ or Musculoskeletal Pain/ or Pain/ 30. Vulvovaginal.mp. 31. gynaecological.mp. 32. weight.mp. 33. Weight gain.mp. or Weight Gain/ 34. "Quality of Life"/ 35. Social function.mp. 36. Cognitive dysfunction.mp. or Cognitive Dysfunction/ 37. fatigue.mp. or Fatigue/ 38. Sleep problems.mp. or Sleep/ 39. insomnia.mp. 40. Depression/ 41. Anxiety/ 42. Mental wellbeing.mp. or Mental Health/ 43. 25 or 26 or 27 or 28 or 29 or 30 or 31 or 32 or 33 or 34 or 35 or 36 or 37 or 38 or 39 or 40 or 41 or 42 44. 4 and 7 and 24 and 43 45. limit 44 to English language |
| Web of Science | Up to 16^th^ March 2020 | 1. “Breast Cancer” 2. “breast neoplasm" 3. "Adjuvant breast cancer" 4. "Breast cancer survivor" 5. #4 OR #3 OR #2 OR #1 6. ("Medication adherence") 7. ("Patient Compliance ") 8. (adher* OR complian*) 9. (persist* OR discont*) 10. #9 OR #8 OR #7 OR #6 11. Tamoxifen 12. Anastrozole 13. Arimidex 14. Exemestane 15. Aromasin 16. Letrozole 17. Femara 18. “Hormonal therapy” OR “hormone therapy” 19. “Aromatase inhibitor” 20. “Endocrine therapy” 21. “Selective estrogen receptor modulators” OR “SERM” 22. “Adjunctive Treatment” OR “Adjuvant Treatment” 23. #22 OR #21 OR #20 OR #19 OR #18 OR #17 OR #16 OR #15 OR #14 OR #13 OR #12 OR #11 24. "adverse effects" OR "Side Effects" 25. "Menopause" or "Hot Flashes" or "Vasomotor Symptoms" 26. Musculoskeletal or "Musculoskeletal Pain" OR "Cancer Pain" or "Low Back Pain" or "Pain" 27. Vulvovaginal OR Gynaecological 28. Weight OR Weight Gain 29. “Quality of Life” 30. “Social Function” 31. “Cognitive dysfunction” 32. Fatigue OR Sleep Problems OR insomnia 33. Depression OR Anxiety OR Mental Wellbeing 34. #33 OR #32 OR #31 OR #30 OR #29 OR #28 OR #27 OR #26 OR #25 OR #24 |
|  |  |  |
